# Uncommon Non-*Candida* Yeasts in Healthy Turkeys—Antimicrobial Susceptibility and Biochemical Characteristic of Trichosporon Isolates

**DOI:** 10.3390/pathogens10050538

**Published:** 2021-04-30

**Authors:** Kamila Bobrek, Ireneusz Sokół, Andrzej Gaweł

**Affiliations:** 1 Department of Epizootiology and Clinic of Bird and Exotic Animals, Wroclaw University of Environmental and Life Sciences, 50-366 Wrocław, Poland; andrzej.gawel@upwr.edu.pl; 2 Private Veterinary Service, SM-ARTVET, 51-361 Wrocław, Poland; irek005@wp.pl

**Keywords:** *Trichosporon*, turkey, yeast-like fungi

## Abstract

The microbiota of the gastrointestinal tract of humans and animals is inhabited by a diverse community of bacteria, fungi, protozoa, and viruses. In cases where there is an imbalance in the normal microflora or an immunosuppression on the part of the host, these opportunistic microorganisms can cause severe infections. The study presented here evaluates the biochemical and antifungal susceptibility features of *Trichosporon* spp., uncommon non-*Candida* strains isolated from the gastrointestinal tract of healthy turkeys. The *Trichosporon coremiiforme* and *Trichosporon (Apiotrichum) montevideense* accounted for 7.7% of all fungi isolates. The biochemical tests showed that *Trichosporon coremiiforme* had active esterase (C4), esterase-lipase (C8) valine arylamidase, naphthol-AS-BI phosphohydrolase, α-galactosidase, and β-glucosidase. Likewise, *Trichosporon montevideense* demonstrated esterase-lipase (C8), lipase (C14), valine arylamidase, naphthol-AS-BI phosphohydrolase, α-galactosidase, and β-glucosidase activity. *T.coremiiforme* and *T. monteviidense* isolated from turkeys were itraconazole resistant and amphotericin B, fluconazole, and voriconazole susceptible. Compared with human isolates, the MIC range and MIC values of turkey isolates to itraconazole were in a higher range limit in both species, while MIC values to amphotericin B, fluconazole, and voriconazole were in a lower range limit. Furthermore, the obtained ITS1—5.8rRNA—ITS2 fragment sequences were identical with *T. coremiiforme* and *T. montevideense* sequences isolated from humans indicating that these isolates are shared pathogens.

## 1. Introduction

The microbiota of the gastrointestinal tract of poultry consists of a diverse collection of bacteria, fungi, protozoa, and viruses which are natural occupants in healthy birds [1]. In cases where there were changes to the host’s immune system, such as an imbalance in the normal microflora, the integrity of the mucocutaneous barrier, a failure to mount a proper immune response or an immunocompromised condition brought on by stress, the opportunistic organisms can cause severe infections [2,3,4].

This is often observed when the microbiota balance is disturbed after an antimicrobial treatment and fungal development is thereby facilitated. Fungal infections in birds should be monitored not only to safeguard poultry health and production, but also in order to protect human health since birds are a reservoir of harmful fungi such as dermatophytes and yeast, which has been confirmed by many research studies [5,6,7].

The best described potential avian sources of pathogenic yeasts are pigeon droppings in which genera *Candida*, *Cryptococcus*, *Rhodotorula*, *Saccharomyces*, *Trichosporon* were commonly identified [8,9,10]. Among birds kept as pets, parrots might be a source of fungi, mainly *Candida* species which could be hazardous to human health, especially to immuno-compromised individuals, children, and the elderly. The most prevalent species were *Candida albicans*, *C. tropicalis*, *C. krusei* and among non-*Candida* species *Trichosporon asteroides* was noted [5,11].

Due to the increased reports of uncommon yeast infections in humans and the antifungal resistance of isolates [12,13,14,15,16], the present study was undertaken to characterize the non-*Candida* fungi isolates from healthy turkeys according to the biochemical and antifungal features of the strains.

## 2. Materials and Methods

### 2.1. Animals and Mycological Investigation

Fungi isolates were taken from 6-week-old British United Turkey (BUT) Big 6 turkeys from 7 flocks—10 birds from each flock reared in Poland [17]. During the rearing period, the birds were monitored for gastrointestinal disorders, and no cases of clinical mycosis were found in the gastrointestinal tract. Additionally, the birds had not been treated with antimicrobials. Swabs (BioMaxima, Lubelskie, Poland) were taken from the beak cavity, crop, and cloaca and cultured on Sabouraud glucose agar with chloramphenicol (Emapol, Warsaw, Poland). Then, they were incubated at 37 °C for 48 h. The negative control was unopened plates taken from batches used for the swab culture. Using classical mycological diagnostic methods, microbiological cultures and microscopic examination, the strains were isolated and identified. Identification of the genera of the fungi was based on the morphological characteristics of the isolates and their growth on CHROMagar media (Emapol, Warsaw, Poland) [17].

### 2.2. Biochemical Analysis of Isolates

The hydrolytic activity of fungi was determined by the API ZYM test (BioMerieux, Marcy-l’Étoile, France) composed of 20 microcupules containing substrates for the evaluation of 19 hydrolytic enzymes: Alkaline phosphatase, esterase, esterase lipase, lipase, leucine arylamidase, valine arylamidase, cystine arylamidase, trypsin, chymotrypsin, acid phosphatase, naphthol-AS-BI phosphohydrolase, α-galactosidase, β-galactosidase, β-glucuronidase, α-glucosidase, β-glucosidase, *N*-acetyl-β-glucosaminidase, α-mannosidase, and α-fucosidase as well as a control. A suspension of fungus cells was prepared from the 24-h culture with the Sabouraud medium, (density—6° according to the McFarland scale) and placed in the microcupules on the API ZYM strip. The results were read according to the instructions provided by the producer.

### 2.3. Antifungal Susceptibility Profile

Yeast inoculum suspensions were prepared as described in the Clinical and Laboratory Standards Institute (CLSI) M27-A3 [18,19]. The inoculum suspensions of fungi isolates were prepared in 0.9% saline solution and adjusted to the turbidity of 0.5 McFarland standard with approximately 1–5 × 10^6^ CFU/mL. This suspension was used directly to inoculate agar plates for the E-test (BioMérieux Polska sp. z o.o., Poland). Quality control was ensured by testing the CLSI recommended strain *C. parapsilosis* ATCC 22019. A purchase was made from BioMérieux Polska sp. z o.o. for E-test strips for amphotericin B (AMB) 0.002–32 μg/mL, itraconazole (ITC) 0.002–32 μg/mL, voriconazole (VOR) 0.002–32 μg/mL, and fluconazole (FLU) 0.016–256 μg/mL). The E-test was carried out according to the manufacturer’s instructions. A RPMI 1640 medium containing 2% glucose (Emapol, Warsaw, Poland) was used for the antifungal susceptibility testing. Each solidified medium was inoculated with a sterile swab dipped into the respective inoculum suspension and then evenly smeared over the surface of the plate. The surface of the agar plate was allowed to dry for 15 min before the E-test strip was placed on it. After drying, the plates were incubated at 35 °C for 48 h. After the plates had been incubated for 24 and 48 h, MICs were read as the lowest drug concentrations at which the border of the ellipse touched the scale on the strip. The final MIC values were based on the consensus between two readers. The minimum inhibitory concentration (MIC) breakpoints are not yet described for *Trichosporon* spp., so the breakpoints recommended for *Candida albicans* as per the CLSI M27-A3 for *Candida* spp. were followed [18,19,20]. The isolate was considered to be susceptible if the MIC value was ≤2 µg/mL for fluconazole, ≤0.125 µg/mL for itraconazole, and ≤0.12 µg/mL for voriconazole. The interpretative criteria for amphotericin B (AMB) were adopted from the literature and the isolates were susceptible if the MIC value was ≤1 µg/mL [20,21,22].

### 2.4. PCR Amplification, Gene Sequencing, and Phylogenetic Analysis

The DNA was isolated using the Genomic Mini AX Yeast (A&A Biotechnology, Gdynia, Poland) according to the manufacturer’s instructions. The general primers ITS1 (5′-TCCGTAGGTGAACCTGCGG-3′) and ITS4 (5′-TCCTCCGCTTATTGATATGC-3′) were used for the ITS1—5.8rRNA—ITS2 fragment amplification [23]. PCR was carried out in a 25 µL reaction mixture containing 50 ng of DNA in 2 µL, 0.25 µL of forward and reverse primer at a concentration of 25 mM, 12.5 µL of PCR Mix Plus (A&A Biotechnology, Gdynia, Poland), and 10 µL DNAse and RNAse water. Amplification was performed in a Bio-Rad T100 PCR Thermal Cycler.

Following an initial denaturation step at 95 °C for 5 min, the reaction mixtures were subjected to 35 cycles of heat denaturation at 95 °C for 30 s, primer annealing at 55 °C for 1 min, and DNA extension at 72 °C for 2 min. Samples were maintained at 72 °C for 10 min for the final extension step. The PCR products were subjected to electrophoresis in a 1.5% agarose gel stained with CybrGreen (Sigma-Aldrich, Poznan, Poland) and visualized under ultraviolet light. The size of the respective PCR products was determined using a molecular mass marker, DNA Marker 1 (A&A Biotechnology, Gdynia, Poland).

The PCR products were isolated from agarose gel using a Gel Out Concentrator Kit (A&A Biotechnology, Gdynia, Poland) and were subsequently sent to Macrogen (Amsterdam, The Netherlands) for Sanger sequencing with the above-mentioned PCR primers (Genomed, Warsaw, Poland). The sequences were analyzed using the Mega 6 software, then compared to sequences from the National Center for Biotechnology Information (NCBI) GenBank database. The sequences were analyzed in comparison with genes sequences of human *Trichosporon* isolates. Moreover, the phylogenetic tree was generated using a MEGA6 software by the neighbor-joining method.

## 3. Results and Discussion

Among the 210 samples taken from healthy turkeys, a total of 26 isolates were identified with the majority (92%) being *Candida* spp., in which two were identified as *Trichosporon* spp. (8%) [17]. Moreover, *Trichosporon* was isolated by other researchers from the intestinal tract of *Gallus gallus*—chickens and hens [6,24,25]. The analysis of metagenomic sequences available in the public bases showed that in poultry the intestinal tract predominates bacteria and fungal species comprising less than 0.1% of the microbiota. In most of the samples in which fungi were detected, the taxonomy analysis showed the presence of *Ascomycota,* to which *Saccharomyces* and *Candida* species belong. The predominance of *Ascomycota* in the chicken gastrointestinal tract was described by Robinsin et al., but in contrast to our own research, the *Basidiomycota* with *Trichosporon* represented 11.7 to 26.8% of the total fungal population [25], while in turkeys it was 8%. In this study, the obtained data from turkey flocks showed a low frequency of *Trichosporon* occurrence, which is lower than in chickens, probably due to the birds species and age. In young chickens, *Trichosporon* was a predominant genus [25], when in older hens the percent of isolates ranged between 0.5 and 10% [6,24], which was similar in turkeys. The PCR amplification of a ITS1-5.8rRNA-ITS2 fragment and sequencing of turkey isolates confirmed growth of the pre-identified *Trichosporon coremiiforme* (MF992258) and *Trichosporon (Apiotrichum) montevideense* (MF992256). Compared to chickens from which *T. moniliiforme* and *T. asahii* were isolated, the *Trichosporon* species found in turkeys are more comparable with hens and pigeons where *Trichosporon coremiiforme* was also isolated [6,10,25]. *Trichosporon (Apiotrichum) montevideense* was not detected in any other poultry species.

**Figure 1:**
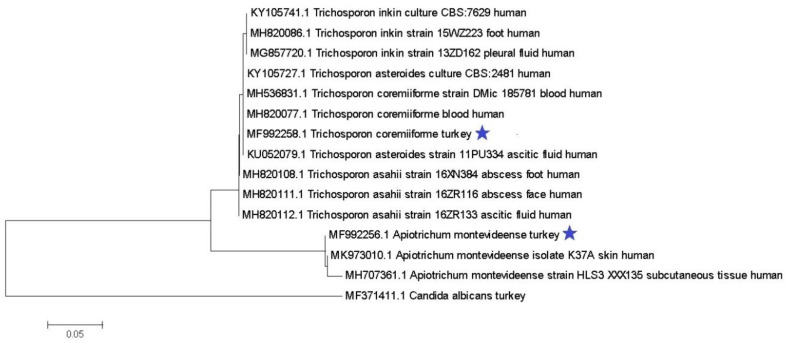
Neighbor-joining tree based on the ITS1-5.8S-ITS2 region. The turkey isolates are marked with an asterisk.

In an attempt to create a phylogenetic relationship of turkey isolates by neighbor-joining, the BLAST programme on the National Center for Biotechnology Information GenBank database was used with *Trichosporon* species isolated from humans. The analysis showed that the obtained turkey sequences were identical with other *Trichosporon coremiiforme* and *Trichosporon (Apiotrichum) montevideense* sequences (Figure 1), of human origin. The ITS regions are recommended as the universal fungal barcode within the species, which may identify the geographic and hosts. The presence of mutations in those regions was able to evaluate the genetic distance between the isolates [26].

Biochemical tests showed that *Trichosporon coremiiforme* demonstrated the activity of esterase (C4), esterase-lipase (C8) valine arylamidase, naphthol-AS-BI phosphohydrolase, α-galactosidase, and β-glucosidase. *Trichosporon montevideense* showed the activity of esterase-lipase (C8), lipase (C14), valine arylamidase, naphthol-AS-BI phosphohydrolase, α-galactosidase, and β-glucosidase activity. It is the first biochemical characterization of non-*Candida* strains isolated from turkeys.

The enzymatic activity of T. *coremiiforme* and T. *montevideense* makes it possible to hydrolase many structures of the cell wall such as esters, lipids, glycolipids, glycoproteins, and oligosaccharides, as well as penetrating the host cell membranes. Features which are under normal conditions beneficial to the host, for example, when fungi in the gastrointestinal tract help digest cellulose, may become dangerous when present in organs and the bloodstream since hemolysins, proteases, and lipases allow for the destabilization of host membranes and the degradation of host connective tissues [27].

For an immunocompromised host aside from enzymatic activity, the resistance of antifungal agents is crucial. If the isolates are resistant to antifungal agents, the infection may be life-threatening for the host. *T.coremiiforme* and *T. monteviidense* isolated from turkeys were susceptible to amphotericin, fluconazole, and voriconazole. Among the triazole derivatives, itraconazole was the least active antifungal agent (Table 1), with both isolates being resistant.

Compared to human isolates, the MIC range (0.125–2 and 0.12–1 μg/mL) and MIC values of turkey isolates to itraconazole were in a higher range limit in both species (1.5 and 1 μg/mL). In contrast to itraconazole, the MIC values of *Trichosporon* isolated from turkeys to amphotericin B, fluconazole, and voriconazole were in a lower range limit. Previously, these non-*Candida* strains of antimicrobial resistance had only been observed in wild birds. Isolates from bird feces were tested for antifungal resistance using a commercial kit that covered 11 frequently employed agents, 144 *Candida, Cryptococcus, Rhodotorula*, and *Trichosporon.* It was reported that 45.8% of the strains were resistant to at least four of the 11 drugs, and 18.1% were resistant to all the antifungals tested [32].

In recent years, interest in the human microbiome has increased, and *Trichosporon* has been recognized as a part of it, but also as an emergent pathogen causing invasive and life-threatening fungal infections [33,34]. Most *Trichosporon* species reside harmlessly as commensals on the skin and in the gastrointestinal tract of healthy individuals and are classic opportunistic pathogens, where they are kept under the watchful eye of the immune system and through interactions with the resident microbiome [27].

Among the most frequently isolated *Trichosporon* species in humans, *T. asahii, T.asteroides*, and *T. inkin* were noted [16,30], while *T. coremiiforme* and *T. montevideense* were rarely isolated. Those two species, isolated from turkeys, were not predominant in human infections. However, more biochemical properties and antimicrobial susceptibility data are needed. To the best of our knowledge, this is the first study characterizing the *Trichosporon* spp. isolated from the turkey intestinal tract.

## 4. Conclusions

The *Trichosporon* species are rarely isolated from the intestinal tract of healthy turkeys. There is no genetic diversity in ITS1-5.8rRNA-ITS2 fragments of *T.coremiiforme* and *T. monteviidense* between the turkey and human strains, but turkey isolates in contrast to human strains are more resistant to itraconazole.

## Figures and Tables

**Table 1:** Antifungal susceptibilities of *Trichosporon* isolates after 48 h of incubation compared to human isolates.

Species (Number of Isolates)	Source	MIC (μg/mL)				Reference
		AMB S ≤1 μg/mL R >1 μg/mL	FLU ≤ 8 μg/mL R ≥64 μg/mL	ITC ≤ 0.125 μg/mL R ≥1 μg/mL	VOR S ≤ 1 μg/mL R ≥4 μg/mL	
***Trichosporon coremiiforme* (1)**	**Turkey**	0.5	1	1.5	0.064	Our research data
*Trichosporon coremiiforme* (1)	Fecal material (human)	0.5	1	0.125	0.5	[3]
*Trichosporon coremiiforme*(1)	Human bloodstream	1	1	0.125	0.03	[12]
*Trichosporon coremiiforme* (1)	N/A (human)	8	0.25	1	-	[13]
*Trichosporon coremiiforme* (1)	Human blood	0.5	4	1	0.06	[15]
*Trichosporon coremiiforme* (6)	Blood and respiratory tract	2–4	0.5–4	-	0.03–0.06	[16]
*Trichosporon coremiiforme* (1)	N/A (human)	0.5	1	2	1	[28]
*Trichosporon coremiiforme* (2)	Urine, subcutaneous abscess	4.0–4.0	2.0–2.0	0.25–1	0.06–0.12	[29]
*Trichosporon coremiiforme* (1)	N/A(human)	0.5	0.5	0.125	0.03	[30]
Human *Trichosporon coremiiforme* MIC range		0.5–8	0.25–4	0.125–2	0.03–1	
***Trichosporon (Apiotrichum) montevideense* (1)**	**Turkey**	0.38	0.75	1	0.064	Our research data
*Trichosporon (Apiotrichum) montevideense* (1)	Ascitic fluid	1	0.5	0.125	0.03	[15]
*Trichosporon (Apiotrichum) montevideense* (1)	Skin	0.12	2	0.25	0.06	[29]
*Trichosporon (Apiotrichum) montevideense* (3)	Bronchial lavage	0.25	1–2	0.06–0.13	0.06–0.13	[31]
Human *Trichosporon (Apiotrichum) montevideense* MIC range		0.12–1	0.5–2	0.06–0.25	0.03–0.13	

AMB: Amphotericin B; FLU: Fluconazole; ITC: Itraconazole; VOR: Voriconazole. Ranges on e-tests: FLU (0.016–256 μg/mL), ITC (0.002–32 μg/mL), VOR (0.002–32 μg/mL), and AMB (0.002–32 μg/mL).

## Data Availability

The data presented in this study are available on request from the corresponding author. The data are not publicly available due to privacy.
